# The stability of a novel 0.035-inch guidewire facilitates endoscopic ultrasound-guided hepaticoduodenostomy

**DOI:** 10.1055/a-2468-6484

**Published:** 2024-12-04

**Authors:** Ritsuko Oishi, Haruo Miwa, Kazuki Endo, Hiromi Tsuchiya, Akihiro Funaoka, Yuichi Suzuki, Shin Maeda

**Affiliations:** 1 26437 Gastroenterological Center, Yokohama City University Medical Center, Yokohama, Japan; 2 Department of Gastroenterology, Yokohama City University Graduate School of Medicine, Yokohama, Japan

Endoscopic ultrasound-guided hepaticoduodenostomy (EUS-HDS) for the right posterior branch is an alternative technique to transpapillary drainage 1 2 . However, insertion of a stent delivery system in EUS-HDS is challenging when the posterior branch and puncture route form an acute angle 3 4 . A novel guidewire (CAPELLA 0.035; Japan Lifeline Co., Ltd, Tokyo, Japan) has the advantage of high seeking ability due to a soft tapered tip, while the stiff shaft facilitates stent delivery ( Fig. 1 ). Additionally, it can be used with most devices designed for 0.025-inch guidewires ( Video 1 ).

**Fig. 1:**
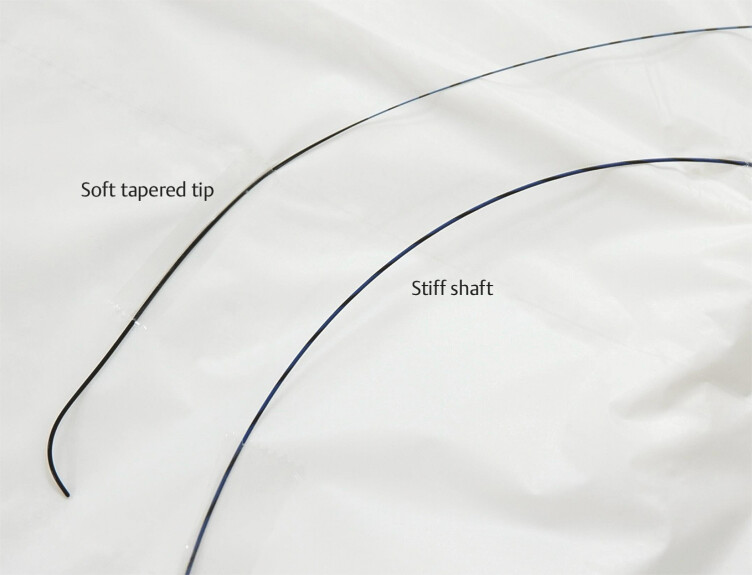
A novel guidewire (CAPELLA 0.035; Japan Lifeline Co., Ltd, Tokyo, Japan) has the advantage of high seeking ability due to a soft tapered tip, while the stiff shaft facilitates stent delivery.

A novel guidewire (CAPELLA 0.035; Japan Lifeline Co., Ltd, Tokyo, Japan) has the advantage of high seeking ability due to a soft tapered tip, while the stiff shaft facilitates stent delivery in endoscopic ultrasound-guided hepaticoduodenostomy.Video 1

A 75-year-old man with Bismuth type IIIa stricture due to intrahepatic cholangiocarcinoma underwent attempted EUS-HDS following failure of transpapillary stenting of the right hepatic duct ( Fig. 2 , Fig. 3 ). The posterior branch was punctured with a 19-gauge needle. After injection of contrast, a 0.025-inch guidewire was inserted into the posterior branch, and the CAPELLA 0.035 was inserted using a double-lumen catheter. The puncture route was dilated using a drill dilator; however, a 5.9-Fr self-expandable metallic stent (SEMS; HANARO Benefit; Boston Scientific, Marlborough, Massachusetts, USA) could not be advanced through the stricture into the bile duct. A tapered balloon catheter also failed to progress into the bile duct, and the 0.025-inch guidewire was dislocated during device exchange. Therefore, an ultra-tapered catheter was carefully inserted into the puncture route over the CAPELLA 0.035. After the guidewire had crossed the stricture, the catheter was reinserted into the bile duct. Although the catheter was bent at an acute angle, CAPELLA 0.035 was smoothly manipulated to advance through the stricture. Subsequently, the guidewire advanced to the duodenum through the papilla, and the SEMS was successfully advanced into the posterior branch over the stiff shaft of the guidewire. Finally, the SEMS was placed from the posterior branch to the duodenum ( Fig. 4 ).

**Fig. 2:**
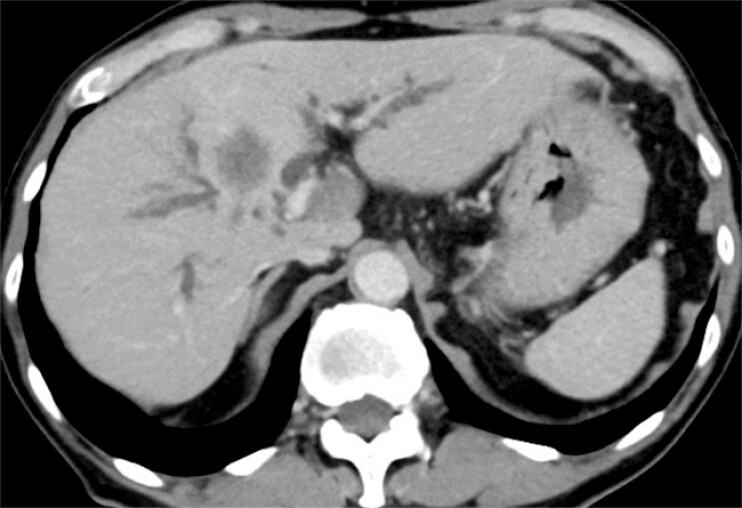
Computed tomography showed a perihilar biliary stricture due to the intrahepatic cholangiocarcinoma.

**Fig. 3:**
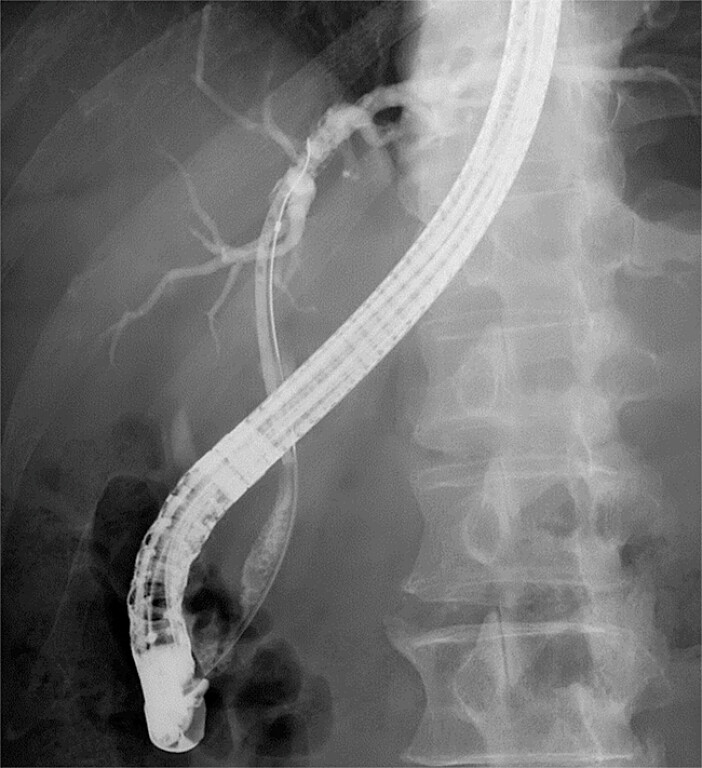
Endoscopic retrograde cholangiopancreatography. A plastic stent was placed in the left hepatic duct; however, a guidewire could not advance into the right hepatic duct.

**Fig. 4:**
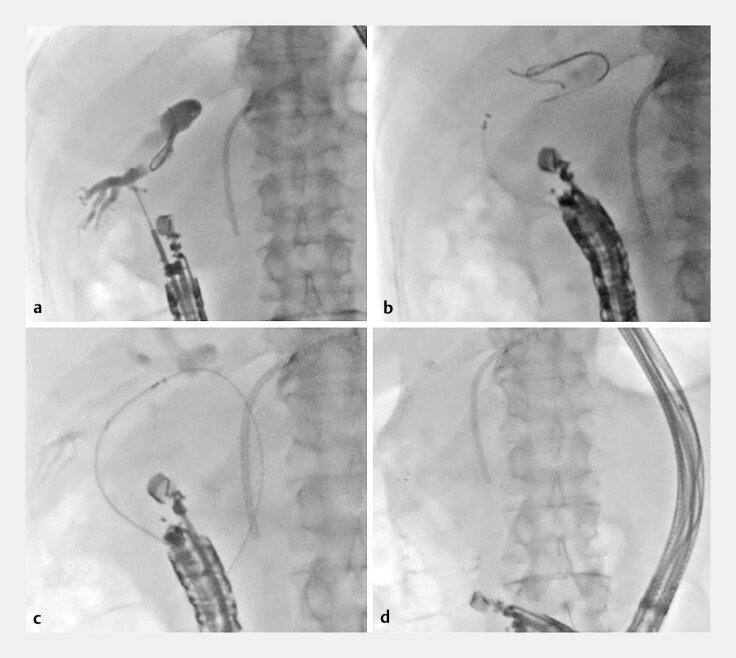
Endoscopic ultrasound-guided hepaticoduodenostomy. **a** The posterior branch was punctured with a 19-gauge needle. **b** A 5.9-Fr self-expandable metallic stent (SEMS; HANARO Benefit; Boston Scientific, Marlborough, Massachusetts, USA) could not be advanced into the bile duct. **c** After placing the CAPELLA 0.035 in the duodenum, the stent delivery system was advanced into the posterior branch. **d** Finally, the SEMS was successfully deployed from the posterior branch to the duodenum.

To the best of our knowledge, this is the first report of SEMS placement in EUS-HDS using the novel 0.035-inch guidewire. The guidewire can offer advantages in interventional EUS.

Endoscopy_UCTN_Code_TTT_1AS_2AH

## Notes

Endoscopy E-Videos https://eref.thieme.de/e-videos
*E-Videos* is an open access online section of the journal *Endoscopy* , reporting on interesting cases and new techniques in gastroenterological endoscopy. All papers include a high-quality video and are published with a Creative Commons CC-BY license. Endoscopy E-Videos qualify for HINARI discounts and waivers and eligibility is automatically checked during the submission process. We grant 100% waivers to articles whose corresponding authors are based in Group A countries and 50% waivers to those who are based in Group B countries as classified by Research4Life (see: https://www.research4life.org/access/eligibility/ ). This section has its own submission website at https://mc.manuscriptcentral.com/e-videos .
